# Increased frequency of social interaction is associated with enjoyment enhancement and reward system activation

**DOI:** 10.1038/srep24561

**Published:** 2016-04-19

**Authors:** Hiroaki Kawamichi, Sho K. Sugawara, Yuki H. Hamano, Kai Makita, Takanori Kochiyama, Norihiro Sadato

**Affiliations:** 1 Graduate School of Human Health Sciences, Tokyo Metropolitan University, Tokyo, 116-8551 Japan; 2 Division of Cerebral Integration, Department of Cerebral Research, National Institute for Physiological Sciences, Okazaki, 444-8585 Japan; 3 School of Medicine, Faculty of Medicine, Gunma University, Maebashi, 371-8511 Japan; 4 Department of Physiological Sciences, SOKENDAI (The Graduate University for Advanced Studies), Hayama, 240-0015 Japan; 5 ATR Brain Activity Imaging Center, Sagara-gun, 619-0288, Japan

## Abstract

Positive social interactions contribute to the sense that one’s life has meaning. Enjoyment of feelings associated through social interaction motivates humans to build social connections according to their personal preferences. Therefore, we hypothesized that social interaction itself activates the reward system in a manner that depends upon individual interaction preferences. To test this hypothesis, we conducted a functional magnetic resonance imaging (fMRI) study in which 38 participants played a virtual ball-toss game in which the number of ball tosses to the participant was either similar to (normal-frequency condition) or higher than (high-frequency condition) the number of tosses to the other players. Participants reported greater-than-anticipated enjoyment during the high-frequency condition, suggesting that receiving a social reward led to unexpected positive feelings. Consistent with this, the high-frequency condition produced stronger activation in the ventral striatum, which is part of the reward system, and the precuneus, representing positive self-image, which might be translated to social reward. Furthermore, ventral striatal activation covaried with individual participants’ preference for interactions with others. These findings suggest that an elevated frequency of social interaction is represented as a social reward, which might motivate individuals to promote social interaction in a manner that is modulated by personal preference.

Positive social connections and harmonious relationships with others contribute to the sense that one’s life has meaning. Therefore, the motivation to form and sustain social connections is one of the most powerful, universal, and influential human drives^1^. Lack of social connection lowers self-esteem^2^ and is a major health risk factor, comparable to smoking and obesity^3^,^4^. In this sense, being with other people and interacting with them in harmonious relationships are fundamental social behaviors.

Social rewards are particularly important motivators for social interaction^5^. One of the most extensively investigated major motives for social interaction is the potential for improving feelings of self-worth and importance through praise and the attention from others^5^. Receipt of both praise and attention activates the ventral striatum, which is part of the reward system, and engagement of the reward system reflects the positive feelings associated with such social rewards^6^,^7^. Therefore, the neural correlates involved in receiving attention from others overlap with the brain regions engaged during pleasure processing.

Because social interaction and harmonious relationships are inherently enjoyable, people might want to interact with others even when the encounter has no purpose other than the interaction itself^8^. Positive stimulation (i.e., enjoying the feelings associated with interpersonal closeness and communion through social interaction) is another major motive for social interaction^5^. Because greater social interaction enhances feelings of interpersonal closeness and communion^9^, the extent of arousal resulting from social interaction *per se* might depend on the quantity of social interactions. However, it is difficult to develop an experimental paradigm that modulates the quantity of social interactions during interactions with others, especially one that increases social interactions, rather than modulating the quality of social interaction (e.g., positive vs. negative social evaluation). Consequently, the neural mechanisms underlying the positive stimulation associated with social interactions are not well understood.

Because social reward might map onto existing structures that register pleasure, these regions might play key roles in building positive social connections^10^. The cortico-basal ganglia circuit, which represents pleasure, is at the heart of the reward system^11^. Key components of this circuit include cortical regions such as the orbitofrontal cortex (OFC) and medial prefrontal cortex (mPFC) and subcortical regions such as the caudate, putamen, and nucleus accumbens, which collectively make up the striatum^12^. The OFC/mPFC may integrate value across different stimuli or stimulus dimensions^13^. Inputs from the mPFC and OFC terminate within subregions of the ventral part of the striatum, where they converge and interweave in a complex manner with projections from other cortical areas^14^. Therefore, the striatum, especially the ventral part of this structure, is modulated by other cortical areas including OFC/mPFC^14^. Via this modulation, the ventral striatum can be tuned for reward-based incentive drive of social behaviors^11^. Accordingly, we hypothesized that increased social interaction would enhance activation in the OFC, mPFC, and/or striatum of the reward system, representing social reward. Furthermore, people differ in regard to their optimal or preferred overall level of interaction^15^; thus, the motivation associated with social interaction might be modulated at an individual level. Because individual preference for social interaction^16^ or belonging to social groups^17^,^18^ modulates the brain response to social interaction, activation of the reward system might also be modulated by individual preferences regarding social interaction. Therefore, if increased social interaction acts as a motivation for social interaction, activation of the reward system (OFC, mPFC, and/or striatum) would be related to an individual’s preference for interaction with others.

The virtual ball-toss game is a widely used experimental paradigm for investigating the psychological constructs^19^ and neural mechanisms^20^ underlying social exclusion resulting from reduced social interaction. This paradigm only requires participants to toss a ball with other players. Even when inclusion in the ball-toss game is paired with a financial loss, participants still felt threatened when they were excluded from the game^21^. In this sense, playing the ball-toss game with other players might be a reward in and of itself that overcomes the penalty of financial loss. Thus, the virtual ball-toss game is a suitable experimental paradigm for investigating the neural correlates underlying modulation of the quantity of social interaction. We predicted that the OFC, mPFC, and/or striatum would be activated during inclusion in the ball-toss game, and that this activation would covary with individual interaction preferences measured using the Collectivism Scale^22^.

## Results

### Questionnaire results

**Figure 1:**
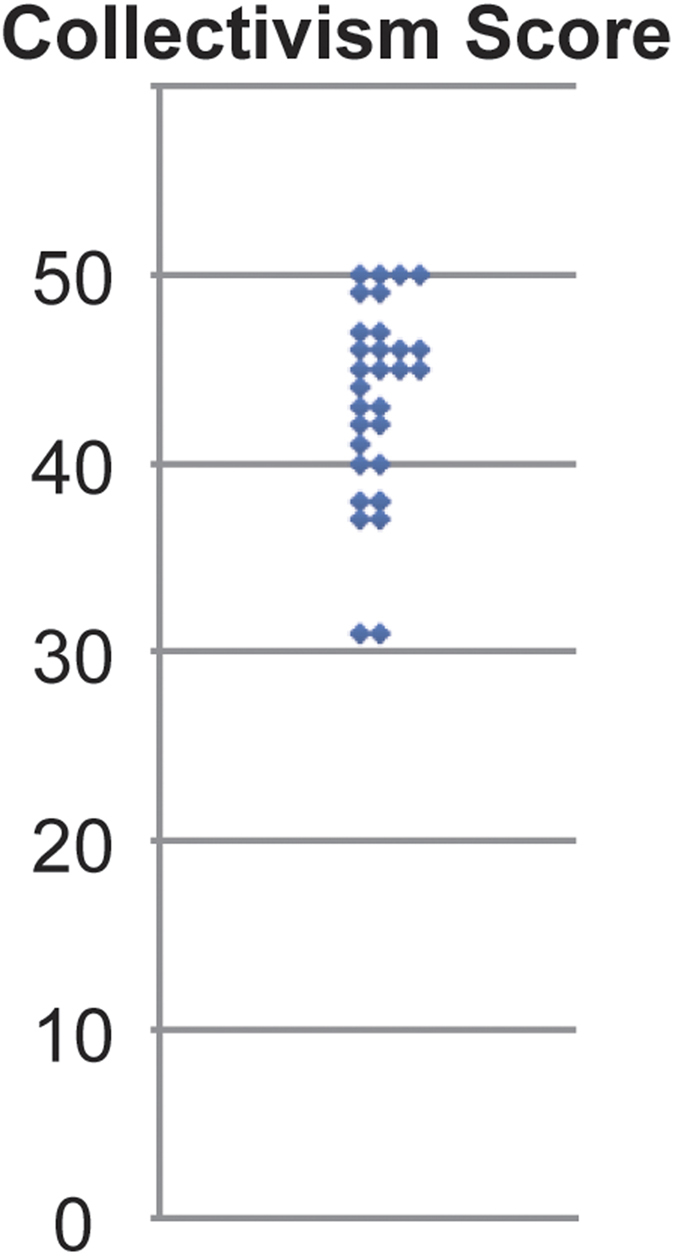
Collectivism scores. Collectivism scores for 31 participants are shown. The minimum and maximum scores of the 31 participants were 31 and 50, respectively.

Among participants, the average score ± standard error of the mean (SEM) on the Collectivism Scale was 43.13 (±0.94) (Fig. 1). This result was similar to a previous study measuring collectivism scores in a group of Japanese participants (average score ± standard deviation = 43.62 ± 7.20)^22^.

#### *Rating scores*

**Figure 2:**
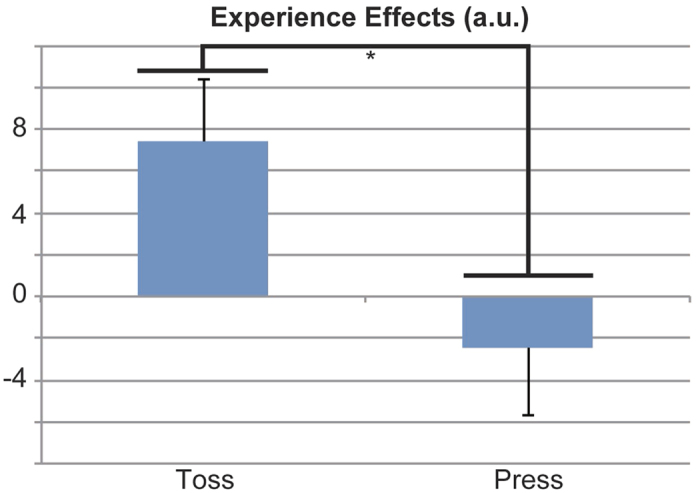
Experience effects of the enjoyment ratings. Experience effects are shown for the ball-toss run (Toss) and the button-press run (Press). Experience effects were calculated as (experience [high-frequency enjoyment – normal-frequency enjoyment] – expectation [high-frequency enjoyment – normal-frequency enjoyment]) for the two runs (ball-toss and button-press). Thus, experience effects ranged from −200 to 200. There was a significant difference between experience effects (*p* = 0.014; paired *t*-test). a.u., arbitrary units.

During the ball-toss runs, the average experience-effect (high-frequency effects of enjoyment rating inside [experience] minus outside [expectation] the fMRI scanner) score (±SEM) was 7.38 (±2.99). By contrast, the average experience-effect score (±SEM) during the button-press runs was 2.43 (±3.24). Paired t-tests revealed a significant difference between experience effects during the ball-toss and button-press runs (*p* = 0.014) (Fig. 2).

### 

### fMRI results

**Figure 3:**
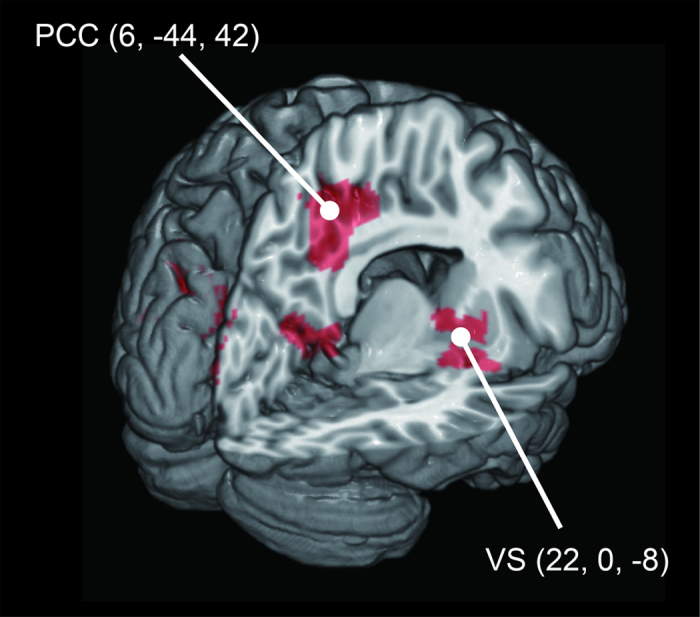
Significant activation of the (ball-toss – button-press) high-frequency effect. Two significant clusters located in the precuneus and right ventral striatum are shown. The activation was thresholded at a voxel-level uncorrected *p* < 0.005 and a cluster level family-wise error (FWE) corrected *p* < 0.05.

**Figure 4:**
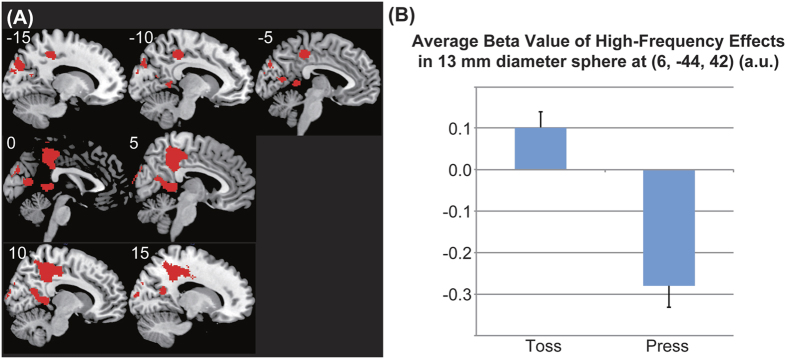
Significant cluster in the precuneus. (**A**) The significant cluster in the precuneus is shown. The activation was thresholded at a voxel-level uncorrected *p* < 0.005 and a cluster level family-wise error (FWE) corrected *p* < 0.05. (**B**) Average beta values in the significant cluster related to the high-frequency effects (high-frequency – normal-frequency) during the ball-toss run (“Toss”) and the button-press run (“Press”) are shown. a.u., arbitrary units.

**Figure 5:**
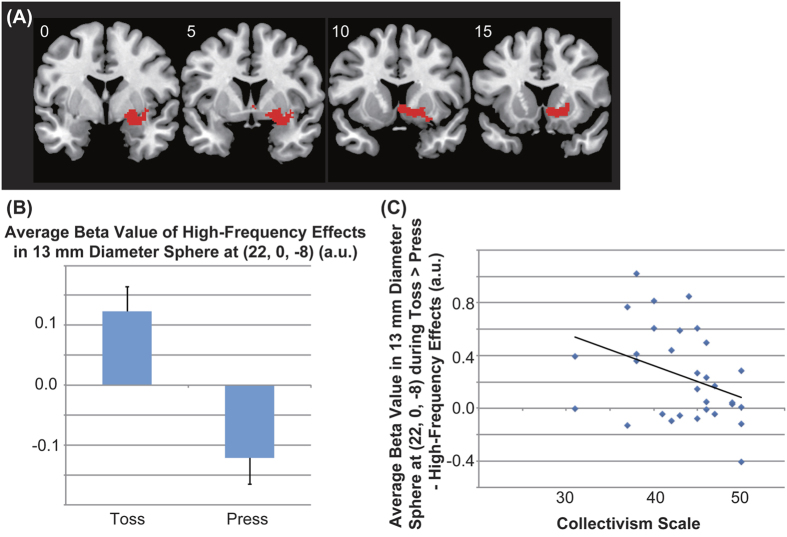
Significant cluster in the right ventral striatum. (**A**) The significant cluster in the right ventral striatum is shown. The activation was thresholded at a voxel-level uncorrected *p* < 0.005 and a cluster level family-wise error (FWE) corrected *p* < 0.05. (**B**) Average beta values in the significant cluster related to the high-frequency effects (high-frequency – normal-frequency) during the ball-toss run (“Toss”) and the button-press run (“Press”) are shown. (**C**) Multiple regression analyses revealed a significant negative correlation between collectivism scores and the average beta value within a 13-mm diameter sphere located at the peak (22, 0, −8) for the (ball-toss [high-frequency – normal-frequency] > button-press [high-frequency – normal-frequency]) (R = −0.361, *p* = 0.046). a. u. means arbitrary unit.

During the ball-toss runs, there were significant high-frequency effects in the precuneus (cluster peak = [6, −44, 42], partly overlapping with occipital cortex and cingulate cortex) and right ventral striatum (cluster peak = [22, 0, −8]) in comparison with the high-frequency effects during the button-press (Participants were only required to press a button by using similar stimuli presentation program to the ball-toss runs except graphical user interface) runs (ball-toss [high-frequency – normal-frequency] > button-press [high-frequency – normal-frequency]) (Table 1 and Figs 3, 4, 5).

Next, we calculated the average beta value in 13-mm diameter spheres located at the peaks of the two significant clusters of each participant for (ball-toss [high-frequency – normal-frequency] > button-press [high-frequency – normal-frequency]). Stepwise multiple regression of the collectivism scores and average beta values within the two spheres revealed a significant negative correlation between collectivism scores and average beta values within the sphere located at the peak of the right ventral striatum cluster (22, 0, −8) (*p* = 0.046; R = −0.361) (Fig. 5C).

Furthermore, in regard to the average beta value in 13-mm diameter spheres located at the peaks of the two significant clusters, we performed *t*-tests between female and male participants to check for possible significant gender effects on activation. No significant differences were detected (precuneus [6, −44, 42]: female = 0.29 [±0.11], male = 0.47 [±0.11], *p* = 0.27; ventral striatum [22, 0, −8]: female = 0.13 [±0.07], male = 0.35 [±0.09], *p* = 0.09), indicating common patterns of activation across genders.

## Discussion

### Experience effects

Forming and maintaining stable interpersonal relationships is a fundamental motivation (i.e., reflecting the need to belong) in human social behaviors^1^. Social interaction is inherently pleasant, and thus plays a key role in forming and maintaining stable relationships. In the high-frequency condition of the ball-toss paradigm, tosses to/from the participants were elevated relative to those among the other players. Consequently, participants found this higher level of social interaction with others more pleasant than the normal-frequency condition. Participants were surprised by the high-frequency social interaction period of the ball-toss game^23^, and hence this condition aroused a relatively large social reward, which in turn led to higher experienced vs. expected enjoyment ratings. Such social rewards caused by increased frequency of social interaction might underpin the sense that one’s life has meaning, emerging from increased feelings of social connection.

### Ventral striatal activation due to high-frequency effects during the ball-toss game

As expected, the task by frequency interaction effects (ball-toss [high-frequency – normal-frequency] > button-press [high-frequency – normal-frequency]) showed significant activation in the right ventral striatum. Because this high-frequency effect was not observed for the high-frequency button-press condition, the striatal activation was due to the increase in social interaction, rather than the increase in button presses.

The striatum is the primary input structure of the basal ganglia^24^, and one of its major roles is reward processing^25^. The ventral striatum represents various types of reward, including abstract rewards, such as the positive feelings triggered by giving^26^,^27^, being actively listened to^7^, receiving praise from others^6^, and monetary gain^6^,^28^,^29^. Furthermore, the ball-toss task involved a simple social behavior without any explicit reward. Thus, the findings suggest that the higher frequency of social interaction was represented as a social reward, as this type of interaction likely fulfills the need to belong^1^.

The enjoyment ratings suggested that the high-frequency ball-toss condition led to a positive experience effect, indicating that the increase in ball tosses to the participant aroused unexpected positive feelings. The ventral striatum, which is activated when a reward is received^30^, represents the actual reward value by evaluating the difference between the experienced reward and the expected reward^31^. This kind of evaluation reflects the temporal difference between the expected and experienced reward during reinforcement learning^32^,^33^. In this study, the ventral striatal activation might reflect the experience of a greater-than-anticipated reward during the ball-toss task. This ventral striatal activation represents the “critic” role in the expected reward^34^, i.e., the unexpected positive feeling aroused by an increase in ball tosses to the participants, which may encourage individuals to engage in social interaction.

In contrast to the observed striatal activation, we did not find any significant activation in mPFC or OFC. Given that the functional boundaries of the human OFC and mPFC are not clearly demarcated^35^, the regions around OFC and mPFC may subserve similar functions, at least to some extent. Although OFC/mPFC may integrate value across different stimuli or stimulus dimensions^13^, a meta-analysis of 27 neuroimaging studies showed that the common currency of reward (primary, monetary, and social) is represented only in the striatum^12^. Several social reward–related paradigms have demonstrated striatal activation but not OFC activation^6^,^7^,^26^, whereas one study reported activation in both OFC and striatum^36^. Another meta-analysis showed that the complexity of the representation and processing of reward covaries with the location of OFC activation (from posterior to anterior)^37^, suggesting that individual differences in the perceived abstractness of social reward (high-frequency social interaction) could decrease the detectability of OFC activation. Therefore, some attributes of social reward related to the present task (such as abstractness) might cause variability in OFC/mPFC activation. In addition, because the primary location of signal loss in echo planar imaging (EPI) gradient-echo sequences is the OFC^38^, signal loss could be another factor that prevented detection of OFC activation in this study.

### Individual differences in ventral striatal activation for high-frequency effects

Scores on the Collectivism Scale were significantly negatively correlated with right ventral striatal activation for (ball-toss [high-frequency – normal-frequency] > button-press [high-frequency – normal-frequency]): participants with higher collectivism traits tended to show lower activation in the right ventral striatum. Individuals with higher collectivism traits perceive the self as interdependent and, thus tend to sustain relationships^39^ by cooperating in groups, even when cooperation comes at some cost^40^. Thus, individuals higher in collectivism tend to form connections between self and other (social interaction). Because approach motivation induced by reinforcing properties drives human behavior toward a positive/desirable event^41^, social interaction *per se* has reinforcing properties. Rewards can be described as their reinforcing properties (i.e., the motivational incentive to engage in specific behavior), based on the expected value of the action’s consequences^12^. Because higher rejection sensitivity is associated with higher scores on the Collectivism Scale^22^ and enhanced striatum activation in the social feedback anticipation phase^42^, higher collectivism traits enhance anticipation of future reward through social interaction. In other words, individuals higher in collectivism expect social interactions to be more highly rewarding. Through repeated experience of social behavior, individuals high in collectivism undergo sufficient reinforcement learning trials^32^,^33^ such that the reward expectation for social interactions becomes similar to the experienced reward. Given that striatal activation negatively covaried with collectivism traits, this activation might reflect a lower value of experienced reward relative to anticipated reward (smaller prediction error) for participants with higher levels of collectivism, due to enhanced and accurate anticipated value of future social reward as a result of prior experience. On the other hand, because individuals lower in collectivism (high individualism) perceive the self as independent of groups and tend to exhibit less cooperation^39^, their social interactions with others are not strongly reinforcing. Thus, individuals lower in collectivism do not have high or accurate expectations of reward for social interaction; consequently, this expectation leads to larger errors in predicting reward value due to lack of experience with social interaction. In line with this consideration, individuals lower in collectivism exhibited relatively high ventral striatum activation. Therefore, an increased frequency of simple social interaction serves as a social reward, and the value of this reward acts as an approach motivation for social interaction in a manner that is modulated by individual preferences for social interaction.

### Advantage of the Cyberball task: detecting the representation of social interaction *per se* in the ventral striatum

The social reward associated with social interaction increases the likelihood that social relationships are considered desirable. Supporting this, social reward-dependence traits in humans are positively correlated with gray-matter density in the ventral striatum^43^. By contrast, previous functional imaging studies did not observe ventral striatal activation during social interactions with other humans^44^,^45^,^46^,^47^, possibly because the experimental tasks had other reward-related characteristics, such as monetary rewards^44^,^45^,^46^ and primary sensory (gustatory) rewards^47^. These reward-related characteristics might interfere with the social reward representation aroused by social interaction *per se* in the ventral striatum. In this sense, the Cyberball paradigm, which does not have reward-related characteristics other than social interaction, is advantageous for investigating the social reward–related activation aroused by social interaction *per se*.

### Precuneus activation due to high-frequency effects during the ball-toss game

The task by frequency interaction effects (ball-toss [high-frequency – normal-frequency] > button-press [high-frequency – normal-frequency]) also showed significant activation in the precuneus. In contrast to ventral striatal activation, precuneus activation was not significantly correlated with scores on the Collectivism Scale. Thus, precuneus activation represents common activation during the processing of social interaction, and was not directly modulated by preference for social interaction.

The precuneus serves a wide range of self-related functions, including episodic memory retrieval and self-processing operations^48^,^49^,^50^. In addition to these task-dependent activations, the precuneus is prominently activated during the resting state (task-independent activation)^51^. In the high-frequency condition, ball-tosses from/to participants were increased in comparison to ball-tosses between two other players. In this sense, the high-frequency condition of the ball-toss run might implicitly remind participants of their social relationships. Through this kind of social interaction including receiving reputation, humans update the self-related image^52^ represented in the precuneus^53^. Furthermore, reputation-based social behavior activates the precuneus^54^. Because the precuneus is functionally connected with the striatum, which is part of the reward system^54^, positive self-image may be translated to social reward represented in the ventral striatum^6^. Consistent with the results of these previous studies, the precuneus activation observed in the current study could reflect common functions involved in representing increased social interaction as a social reward in the ventral striatum.

### Limitations

This study did not measure the expectation of enjoyment during the normal- and high-frequency conditions prior to the fMRI experiment. This was because we did not want the participants to anticipate the experimental manipulation, as this should be avoided in social neuroscience experiments. Therefore, we measured the expectation of enjoyment (social reward) after the fMRI experiment. It is possible that this manipulation confounded our measurements of expectation, because humans anticipate rewards based on previous experience through reinforcement learning^32^,^33^. In such cases, the expectation values should change direction, decreasing the difference between the ratings for expectation and experience. However, the expectation effects during the ball-toss condition were greater than 0, suggesting the presence of expectation effects, especially for the ball-toss conditions.

Although 32 participants appeared to believe the task manipulation (i.e., they believed that they played with real players), as demonstrated by post-experiment interviews, it is possible that the experiment was influenced to some extent by the usage of virtual players. In other words, this task may have implicitly required participants to take a kind of ‘spectator’ view of social interaction^55^. In comparison with social interaction with real players, interaction with virtual players results in less activation in social cognition–related areas such as the superior temporal sulcus^45^. However, even when a participant knows that the other players in a ball-toss game are virtual, they experience an emotional response similar to that resulting from play with real players^56^. In addition, even if this paradigm interferes with detection of brain activity related to quantity of social interaction, we believe that our results showing activation in the ventral striatum and precuneus are robust.

## Conclusions

An increase in toss reception during the ball-toss game acted as a social reward, which was represented by increased activation in the right ventral striatum. Ventral striatal activation accompanied by precuneus activation represents positive self-image, which might be translated to social reward. Furthermore, the right ventral striatal activation was particularly evident in individuals who do not tend to anticipate social reward during social interaction with others. These results indicate that a simple social interaction *per se* is socially rewarding in a manner that is modulated by individual preferences for social interaction. Social reward aroused by social interaction *per se* might increase motivation to interact with others.

## Methods

### Participants

Thirty-eight adults (21 males and 17 females) took part in the experiment. In this study, we sought to investigate neural correlates that are common across gender. Therefore, we recruited comparable numbers of participants of each gender. The average age ± SEM of the participants was 21.24 ± 0.27 years (males, 21.52 ± 0.39 years; females, 20.88 ± 0.38 years). All participants had normal or corrected-to-normal visual acuity, were right-handed according to the Edinburgh handedness inventory^57^, and were free of neurological and medical disorders. The participants received monetary compensation for their time. The protocol was approved by the ethical committee of the National Institute for Physiological Sciences, Okazaki, Japan. The experiments were undertaken in compliance with national legislation and the Code of Ethical Principles for Medical Research Involving Human Subjects of the World Medical Association (Declaration of Helsinki). All participants provided written informed consent.

### Questionnaire

Participants completed the Collectivism Scale^22^, a 14-item scale that measures allocentric tendency. Each item is rated on a five-point scale ranging from 1 “not at all” to 5 “very much”. Higher collectivism scores indicate high allocentric traits in social interaction, which are associated with higher affiliative tendency and higher sensitivity to rejection^22^.

Following the fMRI experiment, outside the scanner, participants rated how much they had expected to enjoy the normal-frequency and high-frequency conditions of the ball-toss and the button-press runs using a visual analog scale (VAS) (ranging from 0 to 100, where 0 indicated “not at all”, and 100 indicated “very much”).

### Presentation of visual stimuli

Visual stimuli were presented using Presentation software 14.4 (Neurobehavioral Systems, Inc.) implemented on a personal computer (dc7900; Hewlett-Packard, Ltd.). A liquid crystal display (LCD) projector (CP-SX12000; Hitachi, Ltd.) located outside and behind the scanner projected the stimuli through a waveguide to a translucent screen, which the participants viewed via a mirror placed in the MRI scanner. The spatial resolution of the projector was 1,024 × 768 pixels, with a 60-Hz refresh rate. The distance between the screen and each participant’s eyes was approximately 175 cm, and the visual angle was 13.8° (horizontal) × 10.4° (vertical). Responses were collected via an optical button box (Current Designs, Inc.).

### Task design

The task design consisted of a ball-toss run and a button-press run. The order of the runs was counterbalanced across participants.

#### *Ball-toss task*

In the ball-toss run, the participants were required to play a virtual ball-toss game^19^,^20^,^58^,^59^ with four same-gender players that were unknown to them. The participants were told that they would be playing a virtual ball-toss game in an fMRI scanner along with four players located in a remote experimental room. After the fMRI experiment, the participants were required to describe any thoughts related to task manipulation. Six participants reported that they suspected that the ball tosses from other players were controlled by a computer program. We interviewed the remaining 32 participants to determine whether they had suspicions about the task manipulation (specifically, whether the ball tosses were controlled by a computer program), and found that all 32 participants appeared to believe that they were actually playing with human participants. In reality, each participant played with four virtual (computer-controlled) players. The participants were fully debriefed about the experiment at the end of the study.

After a 2-min practice run, the participants completed a ball-toss run (5 min 40 s). During the ball-toss game, photographs of four virtual players and the participant were placed at the vertices of a regular pentagon. Twenty-five seconds after the start of fMRI scanning, ball tossing was initiated with equal probability by one of the five players. Participants tossed a ball to one of the other four virtual players by pressing one of four buttons using the right index, middle, ring, and little fingers. The successful ball-toss ratio of each participant (the number of button presses resulting in successful ball tosses relative to the total number of possible ball tosses) was over 90%, indicating that the participants understood and performed the task correctly.

**Figure 6:**
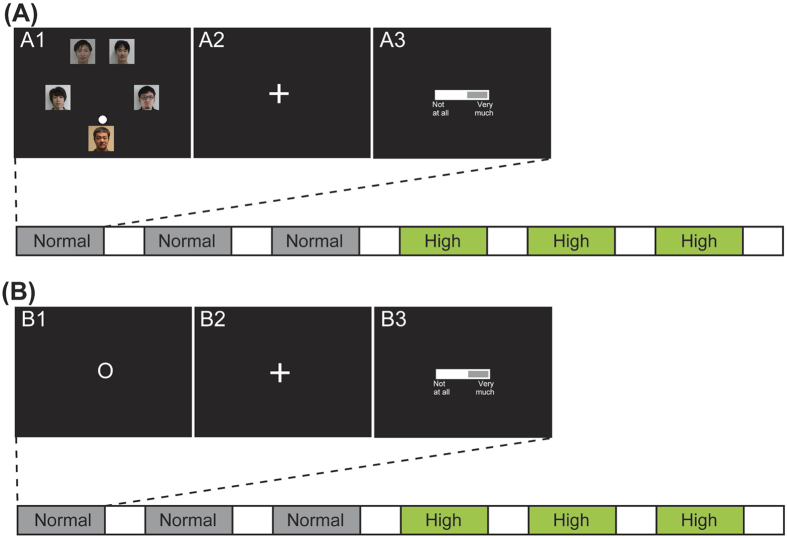
Schematic of the experiment. (**A**) Time course of stimulus presentation in the ball-toss run. Participants were required to toss a ball with the other four players for 30 s (A1). After a fixation cross appeared for 2.5 s (A2), participants were asked to evaluate their level of enjoyment during the preceding ball-toss block for 5 s (A3). This was followed by the presentation of a fixation cross for 15 s. (**B**) Time course of stimulus presentation during the button-press run. Participants were required to press a button when “O” appeared (B1). The button-press block was 30 s. After a fixation cross was presented for 2.5 s (B2), participants were asked to evaluate their level of enjoyment during the preceding button-press block for 5 s (B3). This was followed by the presentation of a fixation cross for 15 s.

There was one run of the ball-toss task, consisting of six ball-toss blocks, six rating blocks, and six rest blocks. In the ball-toss blocks, the participants played the ball-toss game with the four other virtual players for 30 s. There were 30 ball tosses in each block. There were two types of ball-toss block: normal- and high-frequency conditions. In the normal-frequency condition, each participant had six ball tosses during a block. In the high-frequency condition, the number of ball tosses from each participant was 13 per block. Each of the other four players received the ball with equal probability during the normal- and high-frequency conditions. Similar to the experimental design of a previous study^16^, if the target condition (in this study, the high-frequency condition) occurred prior to the normal situation, the participants anticipated a similar pattern in the normal condition, and such anticipation might interfere with investigation of the high-frequency effects. Taking this into account, the high-frequency condition always occurred during the last three blocks of the task. If participants failed to pass the ball within a given time period (1 s), the ball was automatically tossed to another player. After the ball-toss block, the participants were presented with a fixation cross, located at the center of a screen, for 2.5 s. Then, the participants rated how much they enjoyed the prior ball-toss block using a VAS (ranging from 0 to 100, where 0 indicated “not at all” and 100 indicated “very much”). The time period for completing the enjoyment ratings was 5 s. After the rating block, the fixation cross was presented for 15 s (rest block) to allow cerebral blood-flow levels to return to baseline (Fig. 6).

#### *Button-press task*

In the button-press run, the participants were required to press a button according to the following rules. As the stimuli-presentation program was the same except for the graphic user interface, the timing of the button-press cues, the number of button presses, and the condition order were similar to the ball-toss run.

After one 2-min practice run, the participants completed one button-press run (5 min 40 s). During the button-press run, button-press cues (O = “button press” and X = “no button press”) appeared at the center of the screen (Fig. 6). The button-press block started 25 s after the start of fMRI scanning. Participants pressed one of four buttons using the right index, middle, ring, and little fingers. The successful button-press ratio of each participant (the number of button presses performed relative to the number of button press cues) was over 90%, indicating that the participants understood and performed the task correctly.

There was one run of the button-press task, which included six button-press blocks, six rating blocks, and six rest blocks. The button-press blocks were 30 s long. There were two types of button-press block: normal- and high-frequency. The first three button-press blocks were the normal-frequency condition, and the final three button-press blocks were the high-frequency condition. In the normal-frequency condition, there were six button-presses per block, and in the high-frequency condition there were 13 button-presses per block. If participants failed to press a button within 1 s, the button press cue changed to “X”. If participants successfully pressed the button, the inner part of the “O” turned white. After the button-press block, a fixation cross was presented at the center of the screen for 2.5 s. Then, the participants rated their enjoyment during the prior button-press block using the same VAS scale described above. After the 5-s rating block, the fixation cross was presented for 15 s (rest block) to allow cerebral blood-flow levels to return to baseline (Fig. 6).

### fMRI data acquisition

A 3 T scanner (Verio; Siemens, Ltd., Erlangen) was used for acquisition of fMRI data. Each participant’s head was immobilized in a 32-element phased-array head coil. fMRI was performed using an EPI gradient-echo sequence (echo time [TE] = 30 ms; repetition time [TR] = 2,500 ms; field of view [FOV] = 192 × 192 mm^2^; flip angle = 80°; matrix size = 64 × 64; 39 slices; slice thickness = 3 mm; total number of volumes = 136). Whole-brain high-resolution, T1-weighted anatomical MRI using magnetization prepared rapid acquisition gradient echo (MP-RAGE) was also performed for each participant (TE = 2.97 ms; TR = 1,800 ms; FOV = 256 × 256 mm^2^; flip angle = 9°; matrix size = 256 × 256 pixels; slice thickness = 1 mm).

### fMRI data analysis

Functional images were analyzed using SPM8 revision 4667 (The Wellcome Trust Centre for Neuroimaging; http://www.fil.ion.ucl.ac.uk/spm^60^) implemented in MATLAB 2011b (MathWorks, Inc.). The first four volumes of each fMRI run were discarded because the MRI signal was unsteady. After performing motion correction, Fourier phase-shift interpolation was used to correct the slice timing of each image to the middle slice. Then, the mean of the realigned EPI images was co-registered with the T1-weighted MP-RAGE image. Subsequently, the co-registered T1-weighted MP-RAGE image was normalized to the Montreal Neurological Institute (MNI) template using linear and nonlinear three-dimensional (3D) transformations. The parameters from this normalization process were applied to each of the EPI images. Finally, the anatomically normalized EPI images were resampled to a voxel size of 2 mm × 2 mm × 2 mm and spatially smoothed using a Gaussian kernel of 8 mm full width at half-maximum (FWHM). After realignment, head-movement parameters were checked to confirm that none of the runs included head movements over 3 mm.

One participant did not appear to follow the instructions, because she reported the same enjoyment ratings (100) under all conditions. In addition, six participants did not believe the task deception (i.e., they believed they were playing with virtual, computer-controlled players). Therefore, data from these seven participants were excluded, and data were analyzed from the remaining 31 participants (21.10 ± 0.27 years; 16 males, 21.19 ± 0.43 years; 15 females, 21.00 ± 0.43 years).

The task-related activation was evaluated statistically using a general linear model at the individual level to generate contrast images, which were then incorporated into a random-effects analysis at the group level^60^. For individual analyses, five regressors were defined (ball-toss normal-frequency, ball-toss high-frequency, button-press normal-frequency, button-press high-frequency, and rating blocks) and the general linear model included the six motion parameters and a constant term. The duration of each of the task regressors (ball-toss normal-frequency, ball-toss high-frequency, button-press normal-frequency, and button-press high-frequency) was 30 s, and the rating regressor was 5 s. Comparison of ball-toss and button-press runs revealed differences related to visual stimuli (i.e., ball-toss runs were relatively complex and relevant for participants) and response selection (i.e., ball-toss runs implicitly require response selection, taking into consideration the toss course history). In addition, comparison of normal- and high-frequency conditions revealed differences related to number of responses (i.e., the high-frequency condition required more response) and condition order (i.e., the high-frequency condition was presented after the normal-frequency condition). Because a main goal of this study was to investigate neural correlates underlying effects of quantity of social interaction, activation related to visual stimuli and response selection (by comparing normal- and high-frequency conditions) and response execution and condition order effects (by comparing ball-toss and button-press runs) should be canceled out. Based on this comparison, abstraction processes related to visual stimuli and motor functions including response selection and execution could be canceled out. Furthermore, comparison of normal- and high-frequency conditions in the ball-toss run allowed activation commonly related to social interaction (e.g., activation in medial prefrontal cortex) but not modulated by quantity of social interaction to be canceled out. Thus, using the two types of contrast image related to high-frequency effects (ball-toss [high-frequency >normal-frequency] and button-press [high-frequency >normal-frequency]), group analyses were conducted using paired *t* test. The statistical threshold for these analyses was set at an uncorrected *p* < 0.005 at the voxel level with a family-wise error (FWE) corrected *p* < 0.05 at the cluster level.

Stepwise multiple regression was conducted between the collectivism scores and the average beta values related to (ball-toss > button-press) high-frequency effects within 13-mm diameter spheres located at the peaks of the significant clusters identified by the (ball-toss [high-frequency – normal-frequency] >button-press [high-frequency – normal-frequency]) contrast. The aim of this analysis was to investigate which activations explained personal traits related to collectivism. Sphere diameter was determined from final smoothness (x = 12.8 mm; y = 13.1 mm; z = 12.6 mm).

### Performance data analysis

Enjoyment ratings during the high-frequency condition were compared with those during the normal-frequency condition. In this analysis, experience effects were calculated as (experience [high-frequency enjoyment – normal-frequency enjoyment] – expectation [high-frequency enjoyment – normal-frequency enjoyment]) for the two runs (ball-toss and button-press). This calculation was conducted for the following reason: For eliminating task-specific enjoyment effects in the high-frequency condition, enjoyment ratings in the normal-frequency condition were first subtracted from those in the high-frequency condition. Then, because a greater-than-anticipated reward is represented in the brain reward system^34^, the greater-than anticipated reward value (experience effects: experience >expectation) for relative enjoyment of the high-frequency condition was calculated. Then, paired *t*-tests were conducted to test the experience effects between the ball-toss run and the button-press run.

## Additional Information

**How to cite this article**: Kawamichi, H. *et al.* Increased frequency of social interaction is associated with enjoyment enhancement and reward system activation. *Sci. Rep.* **6**, 24561; doi: 10.1038/srep24561 (2016).

## Figures and Tables

**Table 1:** Significant activation for the (toss – button press) high-frequency effect.

	Cluster *p* (FWE)	Cluster size	Location	x	y	z	*t* value
precuneus/cingulate gyrus/occipital cortex	<0.001	3537	cingulate gyrus	6	−44	42	5.07
			precuneus	8	−30	46	4.89
			precuneus	6	−34	46	4.69
			precuneus	2	−34	48	4.43
			precuneus	14	−52	54	4.29
			precuneus	12	−48	52	4.26
			precuneus	12	−46	48	4.24
			precuneus	8	−46	50	4.23
			precuneus	−10	−36	46	4.11
			cingulate gyrus	8	−44	6	4.09
			occipital cortex	28	−58	22	4.04
			occipital cortex	−14	−86	38	4.00
			precuneus	−4	−32	48	3.94
			precuneus	−6	−36	48	3.87
			cingulate gyrus	−2	−68	10	3.78
			cingulate gyrus	18	−42	36	3.78
right ventral striatum	0.031	591	putamen	22	0	−8	4.54
			caudate	6	12	−4	3.72
			caudate	10	12	−6	3.69
			putamen	22	10	−6	3.36
			putamen	34	2	−10	3.35
			putamen	16	6	−8	3.35
			putamen	22	18	0	3.27
			putamen	24	14	0	3.17
			putamen	36	2	−4	2.99
			putamen	36	−2	−6	2.92

Activation was thresholded at an uncorrected *p* < 0.005 at the voxel level and a family-wise error (FWE) corrected *p* < 0.05 at the cluster level. Table shows local maxima (top 16 maxima) more than 4.0 mm apart in each cluster.
